# Early hepatic artery thrombosis treatments and outcomes: aorto-hepatic arterial conduit interposition or revision of anastomosis?

**DOI:** 10.1186/s12893-024-02359-6

**Published:** 2024-02-17

**Authors:** Sahar Sohrabi Nazari, Mohammad Eslamian, Erfan Sheikhbahaei, Hamidreza Zefreh, Mohammad Mehdi Lashkarizadeh, Alireza Shamsaeefar, Kourosh Kazemi, Hamed Nikoupour, Saman Nikeghbalian, Pooya Vatankhah

**Affiliations:** 1 Shiraz Transplant Research Center, Shiraz University of Medical Sciences, 7th Floor, Khalili St, Shiraz, Iran; 2 Isfahan Minimally Invasive Surgery and Obesity Research Center, School of Medicine, Alzahra University Hospital, Isfahan University of Medical Sciences, Isfahan, Iran

**Keywords:** Liver, Liver transplantation, Hepatic artery thrombosis, Biliary complications

## Abstract

**Background:**

Hepatic artery thrombosis (HAT) is one of the critical conditions after an orthotopic liver transplant (OLT) and leads to severe problems if not corrected promptly. However, multiple treatments have been proposed for HAT, in which surgical revascularization with either auto-hepatic conduit interposition (AHCI) or revision of the anastomosis is more familiar indeed indicated for some patients and in specific situations. In this study, we want to evaluate the success and outcomes of treating early HAT (E-HAT), which defines HAT within 30 days after OLT with either of the surgical revascularization techniques.

**Method:**

In this retrospective study, we collected information from the medical records of patients who underwent either of the surgical revascularization procedures for E-HAT after OLT. Patients who needed early retransplantation (RT) or died without surgical intervention for E-HAT were excluded. Demographic data, OLT surgery information, and data regarding E-HAT were gathered. The study outcomes were secondary management for E-HAT in case of improper inflow, biliary complications (BC), RT, and death.

**Results:**

A total of 37 adult patients with E-HAT after OLT included in this study. These E-HATs were diagnosed within a mean of 4.6 ± 3.6 days after OLT. Two patients had their HA revised for the initial management of E-HAT; however, it changed to AHCI intraoperatively and finally needed RT. Two and nine patients from the AHCI and revision groups had re-thrombosis (12.5% vs. 47.3%, respectively, *p* = 0.03). RT was used to manage rethrombosis in all patients of AHCI and two patients of the revision group (22.2%). In comparison to the AHCI, revision group had statistically insignificant higher rates of BC (47.4% vs. 31.2%); however, RT for nonvascular etiologies (12.5% vs. 5.3%) and death (12.5% vs. 10.5%) were nonsignificantly higher in AHCI group. All patients with more than one HA exploration who were in the revision group had BC; however, 28.5% of patients with just one HA exploration experienced BC (*p* < 0.001).

**Conclusion:**

Arterial conduit interposition seems a better approach for the initial management of E-HAT in comparison to revision of the HA anastomosis due to the lower risk of re-thrombosis and the number of HA explorations; indeed, BC, RT, and death remain because they are somewhat related to the ischemic event of E-HAT than to a surgical treatment itself.

## Introduction

Vascular problems are the second main complication after orthotopic liver transplantation (OLT) [1]. Early hepatic artery thrombosis (E-HAT), which is defined as any arterial occlusion within 30 days after OLT, is one of the most dreaded and frequent vascular complications, affecting 2–12% of OLTs [2–4], which can appear with variant presentations, ranging from a clinically silent to acute hepatic necrosis, biliary problems, graft failure, and even death [5]. Retransplantation (RT) with a rate of 50% was declared the standard E-HAT treatment [6]. However, due to insufficient donors, this option could be substituted by other treatments to salvage the graft; depending on early diagnosis, prompt intervention, and careful surgical technique selection and operation [7]. Treatment options for E-HAT may range from conservative treatment and thrombolysis to urgent revascularization with an endovascular approach, surgical revascularization with open thrombectomy and revision of the anastomosis, using other arterial source for anastomosis, aorta-hepatic conduit interposition (AHCI), and finally RT [4, 8, 9]. Whenever proper arterial inflow does not achieve, the AHCI may use instead. In E-HAT management, AHCI may become mandatory in some cases due to the limitations of other treatments. It has been declared that besides the more proper and reliable inflow with AHCI than a revision of the anastomosis, it is the preferred treatment for revascularization in E-HAT due to the lower risk of re-thrombosis [10, 11]; based on the hypothesis that thrombosis formation is the dark side of arterial manipulation, which the risk increases with the anastomosis revision or any similar vascular intervention [12]. These statements need more investigation because, compared to the revision of the anastomosis, AHCI seems more surgically demanding, has a longer duration of operation, and it has some specific complications [10, 13]. Therefore, in this 11-year study, we want to evaluate and compare outcomes and complications of treating E-HAT by either AHCI or revision of the anastomosis.

## Materials & methods

### Study population

**Fig. 1:**
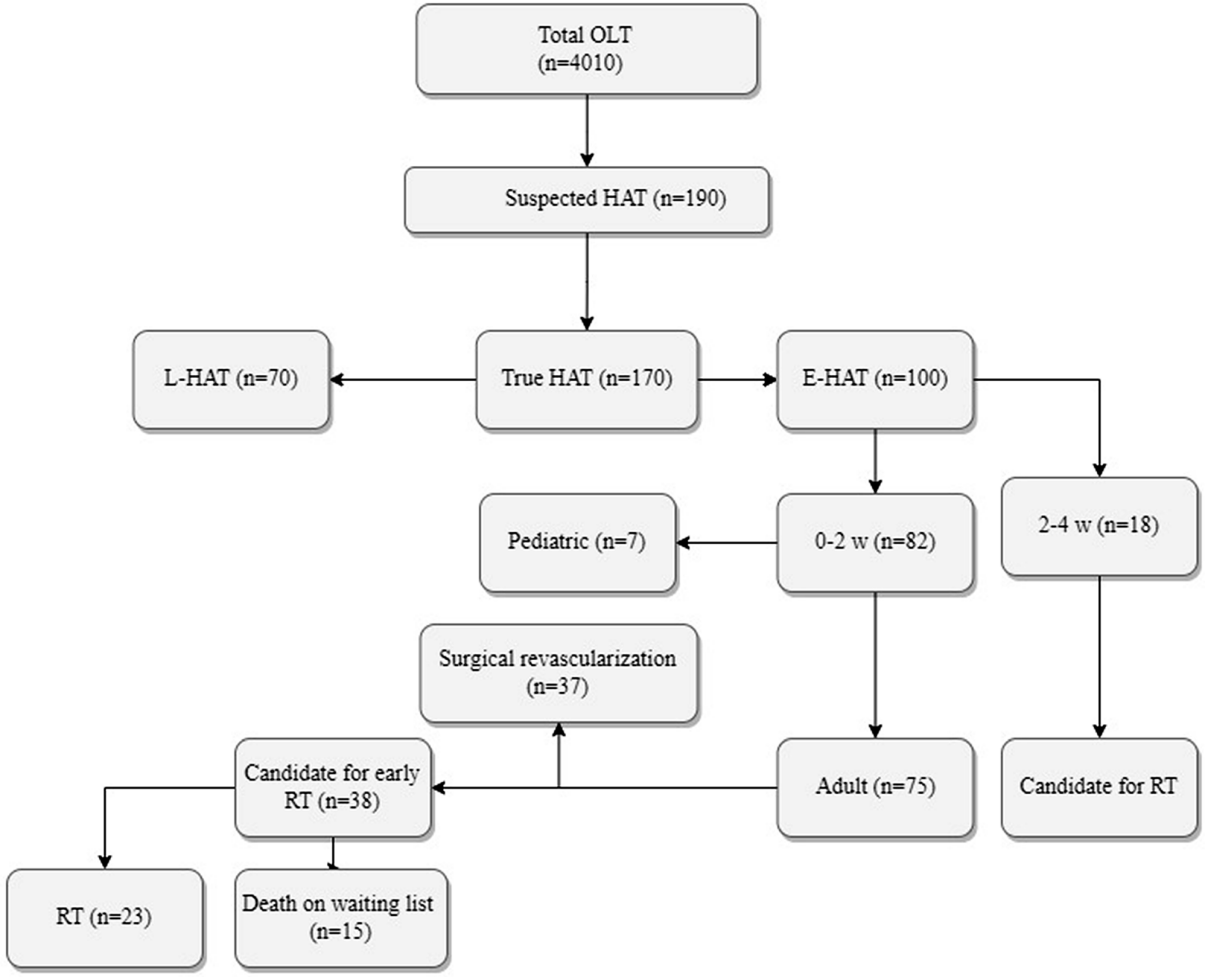
The flowchart of how included patients with E-HAT for this study was found among all of the OLT population of our center (E-HAT: early hepatic artery thrombosis, RT: retransplantation, L-HAT: late hepatic artery thrombosis)

In this retrospective cohort study, we collected information from the medical records of 4,010 patients referred to our high-volume center for OLT in the Middle East. Patients admitted from April 2009 to June 2020 with an impression of E-HAT after OLT (detecting HAT within one month) were included in the study. Pediatric recipients (age < 18 years old), those who underwent early RT for their E-HAT (E-HAT within 2–4 week after OLT and availability of donor) or expired on the waiting list without surgical revascularization were excluded (Fig. 1). Demographic data (age, sex), OLT surgery information (type of liver donation, Model for End-stage Liver Disease score, liver weight, cold ischemic time), and data regarding E-HAT management were gathered. The study outcomes were thrombosis, any biliary complication (BC), and its severity based on the type of treatment, retransplantation, and mortality after surgical revascularization for E-HAT. Documents with more than 20% missing data were excluded.

### Evaluation for potential E-HAT

In our center, doppler US (DUS) is routinely performed intraoperatively and is repeated every 12 h in the first two weeks after OLT to evaluate the hepatic artery blood flow. Moreover, it would be urgently requested in case of any clinical or laboratory derangement. In case of equivocal results, CT-angiography is performed. If there was high suspicion for HAT in any imaging modalities, the patient was transferred to the operating room for HA exploration.

### Surgical technique

In the time of OLT, we usually perform end-to-end donor common HA (CHA) to the recipient CHA anastomosis with 7 − 0 or 8 − 0 proline sutures in an interrupted or continuous manner. The anastomosis technique is chosen based on the characteristics of the artery and surgeon’s preference. As a routine protocol in our center, we usually choose inflow of arterial supply from CHA below the gastroduodenal (GDA) artery by ligating it or use the confluence of the CHA with GDA as a wider patch for a better anastomosis. At the time of exploration for suspected E-HAT, first attempt was revision of the anastomosis if any thrombosis was found at the previous site and proper blood flow in CHA. In case of diminished flow in the recipient CHA, splenic artery was inspected by dissecting the artery at the superior border of the pancreas and then the distal part of the artery is ligated with 2 − 0 silk suture and the proximal part was used as an alternative arterial supply. If splenic artery flow was not satisfactory (e.g., in case of calcification or disseminated arterial intima flap from the CHA to the celiac trunk or thrombosis that was propagated from the CHA to the celiac trunk) an infra-renal aorta-hepatic conduit interposition graft with the procured common iliac artery from the deceased donor was created (graft anastomosed to the aorta with 5 − 0 proline suture in a continuous manner and graft attached to HA with 6 − 0 proline suture in continuous manner). After re-establishing the HA flow, it was ensured by observing the arterial pulsation for a few minutes and intraoperative DUS.

### E-HAT prophylaxis

Post-OLT anticoagulation is not routinely administered in our center for E-HAT prevention. Still, if there was more than one arterial anastomosis or occurring E-HAT, a therapeutic dosage of heparin sulfate (17 unit/kg to reach a 50 s < partial thromboplastin time < 80 s) was administrated after ensuring no active bleeding. After five days, it was changed to acetylsalicylic acid (80 mg daily) [14].

### Postoperative surveillance

After the surgery, the patients were followed by subjective and objective components. A panel of blood parameters including complete blood cell count (CBC), blood urea nitrogen (BUN), creatinine (Cr), aspartate aminotransferase (AST), alanine aminotransferase (ALT), alkaline phosphatase (ALP), gamma-glutamyl transferase (GGT), Albumin (Alb), direct and total bilirubin were checked every week in the first month, every two weeks in the second month and monthly until the first year after OLT. Any symptoms (including fever, jaundice, pruritus, right upper quadrant abdominal pain) or elevation in blood markers would be reassessed with full abdominal and hepatobiliary sonography + DUS and magnetic resonance cholangiopancreatography (MRCP) as needed. Any BC was discussed with a multidisciplinary team. If, based on the investigations, a significant stricture of the bile duct was identified at the anastomosis site, it would be primarily managed with endoscopic retrograde cholangiopancreatography (ERCP) and/or balloon dilatation of the site of stricture and stenting of the common bile duct by an interventional radiologist. Surgical management would be done in cases with unsatisfactory responses by exploring the biliary system and converting duct-to-duct anastomosis to Roux-en-Y hepaticojejunostomy or revising the attachment [10]. Indications of early RT for E-HAT or RT for the next episodes of HAT were simultaneous thrombosis in HA and portal vein, inability to salvage the graft based on intraoperative evidence of irreversible graft damage according to pathologist report of necrosis from frozen section biopsies, disseminated thrombosis to intrahepatic branches, or incapability to use either of the HA anastomosis revision or AHCI for HAT.

### Statistical analysis

Data were analyzed by SPSS software (version 26, IBM Corp. USA). Numerical and categorical variables are presented as median [95% confidence interval for mean] (range is mentioned where needed) and number (percentages), respectively. Distribution of data was checked with Q-Q plot and Kolmogorov-Smirnov or Shapiro-Wilk test. T-test and Pearson chi-square (and Fisher’s Exact Test where needed) were used for statistical analysis of normally distributed numerical (age and liver weight) and categorical variables, respectively and in case of non-normal distribution, Man-Whitney U test was used. Binary regression was used on each dependent variable (Rethrombosis, BC, RT, or death rates) entering all categorical and numerical variables to evaluate the potential predicting factor on each event. A p-value (2-tailed) of < 0.05 was considered a statistically significant level and mentioned where needed.

## Result

**Fig. 2:**
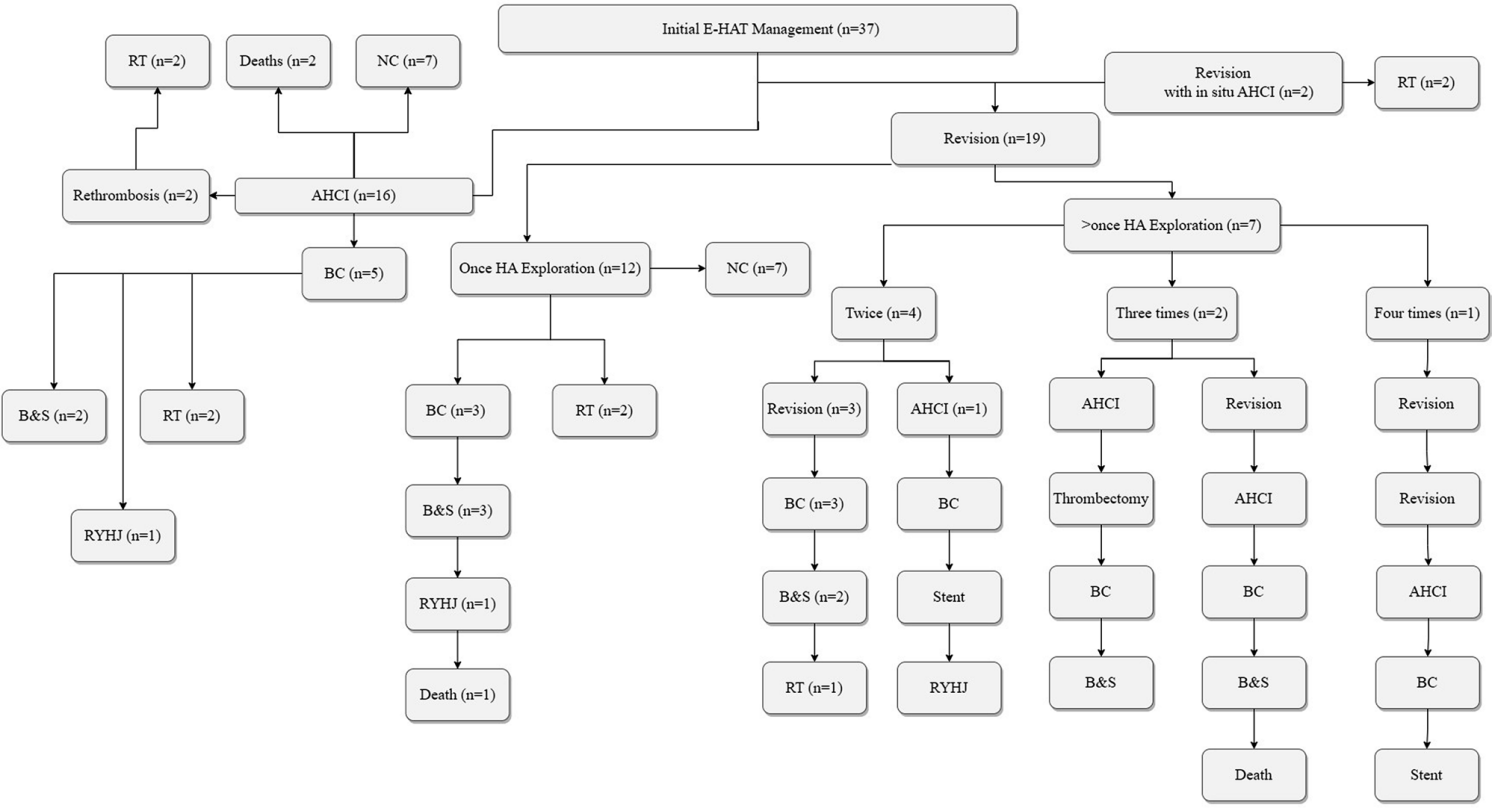
The flowchart diagram of included adult E-HAT patients, their treatments, and outcomes (AHCI: aorto-hepatic conduit interposition, BC: biliary complication, B&S: balloon and stenting, HA: hepatic artery, NC: no complication, RT: retransplantation, RYHJ: Roux-en-Y hepatojejunostomy)

The flowchart of finding those E-HATs that underwent surgical revascularization with either of the treatments in our center is in Fig. 1. E-HAT after OLT was diagnosed within a mean of 4.6 ± 3.6 days, among which three cases were diagnosed intraoperatively. Extracted data, including demographic, OLT indication, surgery information, and laboratory reports are in Table 1. Figure 2 is the management flowchart and outcomes of the 37 adult E-HAT included in the study. Two patients had their HA anastomosis revised for the initial management of E-HAT; however, due to an intimal flap in the HA and its propagation, we changed it to AHCI intraoperatively (i.e., in situ AHCI). Finally, RT (named as early RT due to vascular etiologies) was needed due to disseminated thrombosis into the intrahepatic arterial branches. Considering those patients that did not need any further intervention or special consideration, 7 out of 16 AHCI (43.7%) and 7 out of 19 revision (36.8%) groups were categorized as non-complicated, respectively (*p* > 0.05).

### Rethrombosis

Two and nine patients from the AHCI and revision groups had re-thrombosis (12.5% vs. 47.3%, respectively; OR = 6.3 with 95% CI [1.1–35.7], *p* = 0.03). RT was used to manage the rethrombosis in all patients of AHCI and two patients of the revision group (22.2%). The remaining seven patients in the revision group had their second episode of HAT managed with AHCI (*n* = 2) and re-revision (*n* = 5). Two patients from the re-revision group had another episode of HAT, which was managed with AHCI eventually (Fig. 2).

### Biliary complication, retransplantation, and death

In comparison to the AHCI, patients who had their HA anastomosis revised as their first treatment of E-HAT had statistically insignificant higher rates of BC (47.4% vs. 31.2%); however, RT for nonvascular etiologies (12.5% vs. 5.3%) and death (12.5% vs. 10.5%) were higher in AHCI group but with no statistical significance. All patients with more than one HA exploration observed in the revision group had BC; however, 28.5% of patients with just one HA exploration experienced BC (*p* < 0.001). Regression analysis did not reveal any potential predicting factor on BC, RT, or death. The time to diagnose and treat BC has had a mean of 22.4 ± 20.5 months (range: 4–66, median:10 months). Interval time to RT and death are categorized into early and late groups. Early RT was recorded in six patients with a median of 10.5 days (range: 7–16 days), and early death occurred in two patients within 10 and 15 days after OLT. The three late RT occurred in 480, 630, and 700 days after OLT; late deaths were recorded at 1 and 5 years. All patients with late RT or late death had BC, and the median time between BC diagnosis to RT or death in the late group was ten months (range: 7–47 months).

**Table 1:** Demographic, surgical, and laboratory data of the included adult E-HAT (numerical variables are based on Median [95% confidence interval of mean])

Variable		Total (*n* = 37)	AHCI (*n* = 16)	Revision (*n* = 19) *
Age (years)		45.5 [38.8–47.7]	44 [37–50]	49 [36.6–51]
Male Sex, n (%)		59%	56%	68%
No. of HA Exploration		1 [1.1–1.6]	1	1 [1.1-2.0] **
MELD score		20 [18.3–22.4]	19 [15.8–24.6]	20 [18.5–22.8]
Liver weight (grams)		1150 [1050.9-1234.9]	1090 [945.5–1213]	1160 [1038.5-1329.5]
CIT (min)		480 [370.7-479.2]	450 [328–508]	480 [343.4-508.1]
ALP		244 [243.1-404.5]	198 [151.1-355.2]	331 [239.5-430.1]
Indications of OLT	HBV	13	4	9
	PSC	7	2	5
	HBV + HCC	4	3	1
	AIH	4	3	1
	Wilson	3	1	2
	NASH	2	0	2
	Cryptogenic	2	2	0
	NET	1	1	0
	ALF	1	0	1

AHCI: aorto-hepatic conduit interposition, AIH: autoimmune hepatitis, ALF: acute liver failure, ALP: alkaline phosphatase, CIT: cold ischemic time, HA: hepatic artery, HBV: hepatitis B virus, HCC: hepatocellular carcinoma, MELD: model for end stage liver disease, NASH: non-alcoholic steatohepatitis, NET: neuroendocrine tumor, PSC: primary sclerosing cholangitis, All comparisons were statistically insignificant except for mentioned variable with double stars

*Two patients from the revision group had in situ AHCI and are not included in the analysis

**The HA was explored for thrombosis one, two, three, and four times for 12, four, two, and one patient in the revision group, respectively

## Discussion

The findings of our study indicate that both thrombectomies with (1) the revision of the HA anastomosis in case of having a suitable flow or arterial structure or (2) AHCI in case of not having a proper flow in the HA and splenic artery for revision of the anastomosis are available options for the initial intervention of E-HAT where urgent RT is not feasible. What differentiates these two approaches is related to their major postoperative troubles and how we are going to manage them. Regarding the problems of these procedures, besides HA re-thrombosis, which was significantly higher in the revised approach and exposed patients to more HA exploration and manipulation, both of these treatments have risks of BC, RT, and death. Indeed, we use infra-renal AHCI with procured iliac artery in case of not having a proper flow in the HA and splenic artery or propagation of thrombosis to celiac trunk. Although there are other sources for arterial graft such as GDA, splenic, gastric, colic, or epiploic artery [15–20], our approach of treatment and jump to AHIC is invasive because we couldn’t use the GDA due to previous ligation of its origin from proper HA for a better and easier HA anastomosis and not having a promising flow in the splenic artery. Although previous studies demonstrated their applicability, using other arterial sources need more investigation and experience by surgeon [15–20].

If re-thrombosis was the main factor to determine the success of an E-HAT surgical revascularization approach, Pinna et al. advocated AHCI due to a success rate of 88%, near to our findings. In our study, the AHCI approach, either as an initial choice for E-HAT or secondary after a failed revision, showed some superiority due to much lower rates of early or late re-thrombosis in the HA. Although AHCI is surgically demanding, indicated in specific situations where revision of the anastomosis is not further applicable, and has a longer operation time [21], the direct attachment of a conduit to the aorta or any other similar place provides a high-pressure flow in the HA and creates a more reliable arterial inflow from a large source with lower risk of occlusion due to larger caliber and washing the active molecules [10]. Yet, the AHCI may lead to ischemic-reperfusion injury due to prompt resolution of the obstruction and using a high-pressure flow from the aorta instead of a parallel circulation to the liver, which may cause edema and more liver injury; however, no report of it was found in our study. Secondly, the liver and grafts of this study were prepared for the deceased patient, and using artificial grafts in patients with living-donor OLT may have the same risk of re-thrombosis as the revision, which needs more investigation. Finally, due to the complex pathway of graft from the aorta or any other arterial source to HA, there is a potential risk of external compression from abdominal organs or arterial twisting. However, thrombectomy with the primary revision of the anastomosis is more accessible though it induces more manipulation on the same artery, and this could be why the rate of re-thrombosis and HA exploration was higher in this group. Revision of the anastomosis was successful in 55% of patients in Scarinci et al. study. The etiology of failed revision was not reported, which could be due to re-thrombosis of HA [22]. Re-thrombosis was observed in 12.5% of AHCI and managed with RT; however, late re-thrombosis was not found during our follow-up. Re-thrombosis in the arterial conduit is a potential complication, which may occur in a range of days, as in four patients of Yanaga et al. report to months, as seen in two patients of Pinna et al. study [10, 13]. Graft thrombosis after E-HAT treatment is 16% and 4.2% of re-transplanted cases of E-HAT in the Vivarelli et al. and Muralidharan et al. study [23, 24]. Hypercoagulable diseases such as anti-phospholipid syndrome and paroxysmal nocturnal hemoglobinuria were reasonable explanations for this high rate of re-thrombosis in the Vivarelli et al. report [24]. Most surgeons managed the re-thrombosis after E-HAT with RT [13]; however, due to the shortage in number of donors and prolonged waiting list period, our approach was to reserve RT when damage to the liver is not reversible based on the pathologic report, the prognosis of salvaging the donor’s liver is poor, and revising the anastomosis or AHCI as the final rescue procedure were not applicable such as in disseminated thrombosis into intrahepatic arterial branches. This is why some of our patients who had their HA explored more than once were all from the revision group; all patients of this group faced BC; however, only one needed RT, and one died after all.

The other superiority of AHCI to the revision technique is related to BC, which was significantly associated with the number of HA explorations. Following OLT, the biliary tree is supplied only by HA [8]. Therefore, any vascular compromise leading to biliary ischemia may disrupt the standard structure. This is why biliary necrosis and bile leak are inevitable after E-HAT if not corrected ultimately [25]. This is similar to previous studies indicating that any ischemic event, regardless of the proceeding management, may cause BC after OLT [8, 13, 22, 23]. Based on our findings, all who had more than one HA exploration experienced BC, and all who had their HA revised as the initial treatment approach of E-HAT after OLT. The BC in our AHCI group was higher than Pinna et al. reports, with 17.6% (One biliary leak and two distal CBD strictures) and Park et al. with 28.5% (Five biliary strictures and one leak) [10, 26]. This rate in our revision group was lower than Scarinci et al. [22], with a reported rate of 54%. These indicate that BC is related to the ischemic event and time to manage the E-HAT. Concerning higher rates of re-thrombosis in revision technique and risk of exploring the HA, AHCI seems a better approach, indeed not free of problems, similar to the previous observation [8, 13, 22, 23]. RT and death have been reported in nearly all investigations on E-HAT, indicating that the causality is complicated and multifactorial and cannot be correlated with just a surgical technique [25, 27]. Although the statistical analysis did not find a significant difference, RT and death favor revision more. No RT or death occurred in the AHCI group of the Pinna et al. study, which may explain the low number of E-HAT patients [10]. However, the RT rate after the revision of HA was 36% in Scarinci et al. study, and the death rate after E-HAT management was 21.4% in Wu et al. study [22, 28]. All suggest that every ischemic event insults the liver and increases the chance of graft failure and BC, which indicates an RT subsequently [8] and eventually increases the mortality rate mainly due to biliary sepsis or multiorgan failure [29]. Based on our observation, late RT and death were correlated with BC, and regarding the timing of BC, this could be due to irreversible ischemic intrahepatic cholangiopathy of E-HAT and its thrombosis.

The findings of this study need careful interpretation due to their retrospective nature, heterogeneity in the duration of postoperative follow-up, nonrandom allocation of patients between two groups, and continuing managing the patients based on different surgical indications of each approach; however, these patients were selected from a center of excellence for OLT with a large referral from the whole nation and surrounding countries and with the experienced surgical team.

## Conclusion

Re-thrombosis is a potential complication of manipulating the HA with a rate of 47.3% after revising the hepatic artery anastomosis and 12.5% after aorta-hepatic conduit interposition with procured iliac artery. A higher risk of thrombosis needs more HA exploration and brings more BC. Retransplantation and death are inevitable problems of E-HAT and its surgical revascularization techniques.

## Data Availability

The data will be provided for the secondary analysis from the corresponding author through email address upon reasonable request.

## References

[1] Pareja E, Cortes M, Navarro R, Sanjuan F, López R, Mir J. Vascular complications after orthotopic liver transplantation: Hepatic artery thrombosis. Transplant Proc. 2010;42(8):2970–2. 10.1016/j.transproceed.2010.07.063 20970585

[2] Xue Z, Chen M, Zhang X, Wang G, He X, Wu L (2018). Analysis of early hepatic artery thrombosis after liver transplantation. ANZ J Surg.

[3] Gunsar F, Rolando N, Pastacaldi S, Patch D, Raimondo ML, Davidson B (2003). Late hepatic artery thrombosis after orthotopic liver transplantation. Liver Transpl.

[4] Stange BJ, Glanemann M, Nuessler NC, Settmacher U, Steinmüller T, Neuhaus P. Hepatic artery thrombosis after adult liver transplantation. Liver Transplantation [Internet]. 2003 Jun 1 [cited 2023 Apr 5];9(6):612–20. Available from: https://onlinelibrary.wiley.com/doi/full/10.1053/jlts.2003.50098. 10.1053/jlts.2003.50098 12783404

[5] Puliti Reigada CH, de Ataide EC, de Almeida Prado Mattosinho T, Boin IFSF. Hepatic Artery Thrombosis After Liver Transplantation: Five-Year Experience at the State University of Campinas. Transplant Proc. 2017;49(4):867–70. 10.1016/j.transproceed.2017.01.056 28457413

[6] Bekker J, Ploem S, De Jong KP. Early hepatic artery thrombosis after liver transplantation: a systematic review of the incidence, outcome and risk factors. Am J Transplant [Internet]. 2009 Apr [cited 2023 May 27];9(4):746–57. Available from: https://pubmed.ncbi.nlm.nih.gov/19298450/. 10.1111/j.1600-6143.2008.02541.x 19298450

[7] Stange BJ, Glanemann M, Nuessler NC, Settmacher U, Steinmüller T, Neuhaus P. Hepatic artery thrombosis after adult liver transplantation. Liver Transplantation [Internet]. 2003 Jun 1 [cited 2023 May 27];9(6):612–20. Available from: https://onlinelibrary.wiley.com/doi/full/10.1053/jlts.2003.50098. 10.1053/jlts.2003.50098 12783404

[8] Fujiki M, Hashimoto K, Palaios E, Quintini C, Aucejo FN, Uso TD (2017). Probability, management, and long-term outcomes of biliary complications after hepatic artery thrombosis in liver transplant recipients. Surg (United States).

[9] Kim HB. Urgent revascularization for hepatic artery thrombosis: maybe good for the few, definitely good for the many. 16, Liver Transplantation. 2010. p. 812–4. 10.1002/lt.22115 20583079

[10] Pinna AD, Smith CV, Furukawa H, Starzl TE, Fung JJ, Urgent, revascularization of liver allografts after early hepatic artery thrombosis. Transplantation. 1996 Dec 12;62(11):1584. Available from: 10.1097/00007890-199612150-00010 PMC3018871 8970612

[11] Rogers J, Chavin KD, Kratz JM, Mohamed HK, Lin A, Mark Baillie G et al. Use of autologous radial artery for revascularization of hepatic artery thrombosis after orthotopic liver transplantation: case report and review of indications and options. Elsevier [Internet]. 2001 [cited 2023 May 27];7(10):913–7. Available from: https://www.sciencedirect.com/science/article/pii/S152764650154575X. 10.1053/jlts.2001.26926 11679992

[12] Northup PG, Garcia-Pagan JC, Garcia-Tsao G, Intagliata NM, Superina RA, Roberts LN et al. Vascular Liver Disorders, Portal Vein Thrombosis, and Procedural Bleeding in Patients With Liver Disease: 2020 Practice Guidance by the American Association for the Study of Liver Diseases. Hepatology [Internet]. 2021 Jan 1 [cited 2023 May 27];73(1):366–413. Available from: https://journals.lww.com/hep/Fulltext/2021/01000/Vascular_Liver_Disorders,_Portal_Vein_Thrombosis,.26.aspx. 10.1002/hep.31646 33219529

[13] Yanaga K, Lebeau G, Marsh JW, Gordon RD, Makowka L, Tzakis AG et al. Hepatic Artery Reconstruction for Hepatic Artery Thrombosis After Orthotopic Liver Transplantation. 10.1001/archsurg.1990.01410170076016 PMC3016877 2331222

[14] Kirchner VA, Imber C, McCormack L, Northup PG, Song GW, Spiro M et al. Low-dose aspirin confers protection against acute cellular allograft rejection after primary liver transplantation. journals.lww.com [Internet]. 2022 Oct 1 [cited 2023 Dec 5];36(10). Available from: https://journals.lww.com/lt/abstract/9000/Low_dose_aspirin_confers_protection_against_acute.98994.aspx.

[15] Akbulut S, Kutluturk K, Yilmaz S (2021). Hepatic artery reconstruction technique in liver transplantation: experience with 3,000 cases. Hepatobiliary Surg Nutr.

[16] Yilmaz S, Kutluturk K, Usta S, Akbulut S (2022). Techniques of hepatic arterial reconstruction in liver transplantation. Langenbecks Arch Surg.

[17] Otan E, Akbulut S, Yilmaz S (2021). How to reduce and manage hepatic arterial complications in living and deceased donor liver transplantations. Hepatobiliary Surg Nutr.

[18] Yilmaz S, Akbulut S, Usta S, ... CKL, 2022 undefined. Distal gastroduodenal arterial inflow as a salvage strategy for extensive intraoperative arterial dissect?on in living donor liver transplantation. journals.lww.com [Internet]. [cited 2024 Jan 9]; Available from: https://journals.lww.com/lt/Fulltext/2022/02000/Distal_Gastroduodenal_Arterial_Inflow_as_a_Salvage.27.aspx. 10.1002/lt.26304 34536968

[19] Yilmaz S, Akbulut S, Kutluturk K, ... SDL, 2021 undefined. Splenic artery transposition for hepatic artery reconstruction during liver transplantation: is it the best choice for adequate arterial inflow in extraordinary conditions? journals.lww.com [Internet]. [cited 2024 Jan 8]; Available from: https://journals.lww.com/lt/Fulltext/2021/06000/Using_the_Recipient_s_Left_Gastric_Artery_for.19.aspx. 10.1002/lt.25884 32894802

[20] Yilmaz S, Akbulut S, Kutluturk K, ... SDL, 2021 undefined. Splenic artery transposition for hepatic artery reconstruction during liver transplantation: is it the best choice for adequate arterial inflow in extraordinary conditions? journals.lww.com [Internet]. [cited 2024 Jan 8]; Available from: https://journals.lww.com/lt/Fulltext/2021/04000/Splenic_Artery_Transposition_for_Hepatic_Artery.21.aspx. 10.1002/lt.25884 32894802

[21] Song S, Kwon CHD, Moon HH, Lee S, Kim JM, Joh JW et al. Single-Center Experience of Consecutive 522 Cases of Hepatic Artery Anastomosis in Living-Donor Liver Transplantation. Transplant Proc. 2015;47(6):1905–11. 10.1016/j.transproceed.2015.06.014 26293071

[22] Scarinci A, Sainz-Barriga M, Berrevoet F, Van Den Bossche B, Colle I, Geerts A (2010). Early arterial revascularization after hepatic artery thrombosis may avoid graft loss and improve outcomes in adult liver transplantation. Transpl Proc.

[23] Muralidharan V, Imber C, Leelaudomlipi S, Gunson BK, Buckels JAC, Mirza DF (2004). Arterial conduits for hepatic artery revascularisation in adult liver transplantation. Transpl Int.

[24] Vivarelli M, Barba G, La, Legnani C, Cucchetti A, Bellusci R, Palareti G et al. Repeated graft loss caused by recurrent hepatic artery thrombosis after liver transplantation. Elsevier [Internet]. 2003 Jun 1 [cited 2023 May 27];9(6):629–31. Available from: https://www.sciencedirect.com/science/article/pii/S1527646503000686. 10.1053/jlts.2003.50082 12783408

[25] Wells MM, Croome KP, Boyce E, Chandok N. Roux-en-Y choledochojejunostomy versus duct-to-duct biliary anastomosis in liver transplantation for primary sclerosing cholangitis: A meta-analysis. Transplant Proc. 2013;45(6):2263–71. 10.1016/j.transproceed.2013.01.066 23953538

[26] Park GC, Moon DB, Kang SH, Ahn CS, Hwang S, Kim KH (2019). Overcoming hepatic artery thrombosis after living donor liver transplantations: an experience from asan medical center. Ann Transpl.

[27] Obed M, Othman MI, Siyam M, Hammoudi S, Jarrad A, Bashir A (2021). Early hepatic artery thrombosis after living donor liver transplant: a 13-year single-center experience in Jordan. Exp Clin Transplant.

[28] Wu L, Zhang J, Guo Z, Tai Q, He X,... WJ...: OJ of the, et al. Hepatic artery thrombosis after orthotopic liver transplant: a review of the same institute 5 years later. europepmc.org [Internet]. [cited 2023 May 27]; Available from: https://europepmc.org/article/med/21649568. 21649568

[29] Mourad MM, Liossis C, Gunson BK, Mergental H, Isaac J, Muiesan P (2014). Etiology and management of hepatic artery thrombosis after adult liver transplantation. Liver Transpl.

